# Shotgun metagenomic dataset of surface microbiomes at a train station in Shinagawa, Tokyo

**DOI:** 10.1186/s12863-026-01451-5

**Published:** 2026-06-12

**Authors:** Xinxin Gao, Ayaka Sanui, Dewa A. P. Rasmika Dewi, Alexander G. Lucaci, Christopher E. Mason, Haruo Suzuki

**Affiliations:** 1 Institute for Advanced Biosciences, Keio University, Tsuruoka, Yamagata Japan; 2 Systems Biology Program, Graduate School of Media and Governance, Keio University, Fujisawa, Japan; 3 Faculty of Environment and Information Studies, Keio University, Fujisawa, Kanagawa Japan; 4 School of Public Health, Faculty of Medicine, Nursing, and Health Sciences, Monash University, Melbourne, Australia; 5 Department of Systems and Computational Biomedicine and WorldQuant Initiative, Weill Cornell Medicine, New York, NY USA; 6 The HRH Prince Alwaleed Bin Talal Bin Abdulaziz Alsaud Institute for Computational Biomedicine, Weill Cornell Medicine, New York, NY USA; 7 The Trivedi Institute of Space and Global Biomedicine, Pittsburgh, PA USA

**Keywords:** Urban microbiome, Shotgun metagenomics, Public transit, Surface microbiome, Tokyo

## Abstract

**Objectives:**

The urban microbiome is a significantly underexplored ecosystem which contributes to the health and resilience of the human population and less is known about the microbiome of urban transportation systems that commuters interact with daily. Shotgun metagenomic sequencing data from swab samples were collected at a representative medium-scale urban commuter railway station in Tokyo, Japan, with daily passenger volumes on the order of tens of thousands, in October 2021. The dataset was generated as part of the nationwide “Urban Microbiomes in Japan” project and provides a resource for comparative analyses of urban microbial diversity and future public health surveillance studies in urban environments.

**Data description:**

Three surface swab samples were collected in October 2021 from concrete floor areas near ticket gates at a major railway station in Shinagawa, Tokyo. Samples were collected using Isohelix swabs with DNA/RNA Shield stabilization solution. Metagenomic DNA was extracted and subjected to shotgun sequencing, generating 2 × 150 bp paired-end reads.

## Objective

Recent studies of urban and built environment microbiomes have expanded understanding of microbial diversity in cities [1, 2], revealing both shared and distinct features in taxonomic composition, functional potential, and antimicrobial resistance [3, 4]. Public transportation systems, with high human density and frequent surface contact, serve as important ecological interfaces that facilitate microbial exchange [5, 6]. Previous studies have mainly focused on large transportation hubs [7, 8, 9] or international airports [3], which primarily serve long-distance travellers.

In this study, we investigated a medium-scale commuter train station in Shinagawa, Tokyo, Japan, which primarily serves local residents. This everyday transit environment has received little attention in microbiome research. We present a shotgun metagenomic dataset from concrete floor surfaces near ticket gates, collected in October 2021. This surface type was selected because prior research [10, 11, 12] has demonstrated that among various transit surface types, floor surfaces generally harbor high microbial diversity. This period corresponded to a specific epidemiological context in Japan [13]. This dataset may provide a useful reference for comparative analyses of urban microbial diversity and may support future studies on public health surveillance in urban environments.

## Data description

### Setting and sample collection

Samples were collected in October 2021 in Japan. The site is a medium-scale commuter station serving approximately 140,000 passengers daily, located in Shinagawa City, southern Tokyo (population 422,488 in 2020) [14, 15, 16]. Concrete floor surfaces in front of the ticket gates for three transit lines were swabbed for 3 min each using Isohelix swabs (Cell Projects Ltd., Maidstone, UK) prefilled with 400 µL DNA/RNA Shield medium (Zymo Research, CA, USA). An overview of the deposited data files/data sets is provided in Table 1. Sample metadata—including collection date, location, surface type, and ambient temperature/humidity—were recorded using KoBoToolbox (https://www.kobotoolbox.org/*).* DNA concentration, raw read counts, and accession numbers were subsequently added to the metadata file deposited in Zenodo (Table 1, Data file 1) [26]. Sampling followed the Standard Operating Procedure (SOP) of the MetaSUB consortium [4, 17, 18].

### DNA extraction and sequencing

After sampling in October 2021, samples were transported at ambient temperature to the laboratory. The tubes were prefilled with DNA/RNA Shield (Zymo Research), which stabilizes nucleic acids without immediate refrigeration. Upon arrival, samples were immediately stored at − 80 °C until DNA extraction. Total genomic DNA was extracted in December 2021 using the ZymoBIOMICS DNA Miniprep Kit (Zymo Research Co., CA, USA), following the manufacturer’s instructions. DNA concentration and purity were measured using a Qubit 4 Fluorometer (Thermo Fisher Scientific, MA, USA).

The extracted DNA was subsequently shipped under chilled conditions to Genome Lead Co., Ltd. (Kagawa, Japan). Shotgun metagenomic libraries were prepared using the MGIEasy FS DNA Library Prep Set (MGI Tech Co., Ltd., Shenzhen, China) according to the manufacturer’s PCR-based protocol, with 10 cycles of PCR amplification. Sequencing was performed on a DNBSEQ-G400RS platform (MGI Tech Co., Ltd.) using the DNBSEQ-G400RS High-throughput Sequencing Set, generating 2 × 150 bp paired-end reads for each sample. Sequencing was completed by February 2022, meaning that the time from sample collection to the completion of sequencing was approximately four months.

### Data processing and availability

No pre-processing (such as quality filtering, adapter trimming, or host read removal) was applied prior to data deposition. All raw FASTQ files were deposited in the DNA Data Bank of Japan (DDBJ) Sequence Read Archive (DRA) under BioProject accession PRJDB14136 [19]. The three run accessions are DRR417937 [20], DRR417938 [21], and DRR417923 [22]. Sample metadata, environmental conditions, DNA concentration, raw read counts, and run accession numbers are provided in the metadata file deposited in Zenodo (Table 1, Data file 1) [26].

**Table 1:** Overview of deposited data files/data sets associated with this study

Label	Name of data file/data set	File types (file extension)	Data repository and identifier (DOI or accession number)
Data set 1	DNBSEQ-G400 paired end sequencing of SAMD00520080	FASTQ (.fastq.gz)	DDBJ Sequence Read Archive (DRA): https://identifiers.org/insdc.sra:DRR417937 [20]
Data set 2	DNBSEQ-G400 paired end sequencing of SAMD00520081	FASTQ (.fastq.gz)	DDBJ Sequence Read Archive (DRA): https://identifiers.org/insdc.sra:DRR417938 [21]
Data set 3	DNBSEQ-G400 paired end sequencing of SAMD00520082	FASTQ (.fastq.gz)	DDBJ Sequence Read Archive (DRA): https://identifiers.org/insdc.sra:DRR417923 [22]
Data file 1	sample_metadata_and_sequencing_summary_for_a_train_station_in_Shinagawa_Tokyo.csv	CSV (.csv)	Zenodo: 10.5281/zenodo.19513161 [26]

## Limitations

This dataset has several limitations. First, the number of samples is limited (*n* = 3). Second, sampling was conducted at a single time point. Third, only concrete floor surfaces were sampled. Finally, no pre-processing was applied prior to data deposition.

## Data Availability

The data described in this Data Note are freely and openly accessible in the DNA Data Bank of Japan (DDBJ) and Zenodo. The raw sequencing data are available in the DDBJ Sequence Read Archive (DRA) under the accession numbers DRR417937 [20], DRR417938 [21], and DRR417923 [22]. The associated BioProject accession is PRJDB14136 [19], and the corresponding BioSample records are available under the accession numbers SAMD00520080 [23], SAMD00520081 [24], and SAMD00520082 [25]. Sample-level metadata, environmental parameters, and sequencing summary are available in Zenodo [26]. Please see Table 1 for an overview of all deposited data files/data sets.

## Abbreviations

bp: Base pairs
DDBJ: DNA Data Bank of Japan
DRA: DDBJ Sequence Read Archive
PCR: Polymerase chain reaction
SOP: Standard Operating Procedure
